# A *de novo* loss-of-function *GRIN2A* mutation associated with childhood focal epilepsy and acquired epileptic aphasia

**DOI:** 10.1371/journal.pone.0170818

**Published:** 2017-02-09

**Authors:** Kai Gao, Anel Tankovic, Yujia Zhang, Hirofumi Kusumoto, Jin Zhang, Wenjuan Chen, Wenshu XiangWei, Gil H. Shaulsky, Chun Hu, Stephen F. Traynelis, Hongjie Yuan, Yuwu Jiang

**Affiliations:** 1 Department of Pediatrics and Pediatric Epilepsy Center, Peking University First Hospital, Beijing, China; 2 Department of Pharmacology, Emory University School of Medicine, Atlanta, GA, United States of America; 3 Department of Neurology, Xiangya Hospital, Central South University, Changsha, China; 4 Center for Functional Evaluation of Rare Variant (CFERV), Emory University School of Medicine, Atlanta, GA, United States of America; 5 Center of Epilepsy, Beijing Institute for Brain Disorders, Beijing, China; Aix-Marseille Universite, FRANCE

## Abstract

**Objective:**

N-methyl-D-aspartate receptors (NMDAR) subunit *GRIN2A*/GluN2A mutations have been identified in patients with various neurological diseases, such as epilepsy and intellectual disability / developmental delay (ID/DD). In this study, we investigated the phenotype and underlying molecular mechanism of a *GRIN2A* missense mutation identified by next generation sequencing on idiopathic focal epilepsy using *in vitro* electrophysiology.

**Methods:**

Genomic DNA of patients with epilepsy and ID/DD were sequenced by targeted next-generation sequencing within 300 genes related to epilepsy and ID/DD. The effects of one missense *GRIN2A* mutation on NMDAR function were evaluated by two-electrode voltage clamp current recordings and whole cell voltage clamp current recordings.

**Results:**

We identified one *de novo* missense *GRIN2A* mutation (Asp731Asn, GluN2A(D731N)) in a child with unexplained epilepsy and DD. The D731N mutation is located in a portion of the agonist-binding domain (ABD) in the GluN2A subunit, which is the binding pocket for agonist glutamate. This residue in the ABD is conserved among vertebrate species and all other NMDAR subunits, suggesting an important role in receptor function. The proband shows developmental delay as well as EEG-confirmed seizure activity. Functional analyses reveal that the GluN2A(D731N) mutation decreases glutamate potency by over 3,000-fold, reduces amplitude of current response, shortens synaptic-like response time course, and decreases channel open probability, while enhancing sensitivity to negative allosteric modulators, including extracellular proton and zinc inhibition. The combined effects reduce NMDAR function.

**Significance:**

We identified a *de novo* missense mutation in the *GRIN2A* gene in a patient with childhood focal epilepsy and acquired epileptic aphasia. The mutant decreases NMDAR activation suggesting NMDAR hypofunction may contribute to the epilepsy pathogenesis.

## Introduction

N-methyl-D-aspartate receptors (NMDARs), ligand-gated cation channels, mediate the slow component of excitatory synaptic transmission [1]. NMDARs are heterotetramers composed of two glycine-binding GluN1 subunits and two glutamate-binding GluN2 subunits [1]. Binding of both agonists is required for activation and results in a conformational change leading to the opening of a cation-selective transmembrane pore that catalyzes an influx of calcium and sodium at resting potentials [2,3]. The GluN1 subunit is expressed ubiquitously throughout the brain, whereas the expression of four GluN2 subtypes (A-D) varies spatially and temporally. Messenger RNA for GluN2A and GluN2C is expressed after birth, and their expression levels appear to increase with age. By contrast, GluN2B and GluN2D subunits are expressed prenatally at an early stage of life, and decreases in most brain areas with age [4]. NMDARs play important roles not only in normal brain function, including learning, memory, synaptic plasticity, motor and sensory processes, and nervous system development, but also in a wide range of neurological diseases, such as epilepsy, Huntington’s disease, and Parkinson’s disease, Alzheimer’s disease, autism, and schizophrenia [5–12].

Recently, pathogenic NMDARs mutations have been identified in epilepsy, developmental delay, intellectual disability, autism, attention deficit hyperactivity (ADHD), and schizophrenia [13–20]. The *GRIN2A* gene encoding the GluN2A subunit has been suggested to constitute a locus for mutations in a subset of patients with early-onset seizures [19]. Several case-control studies have identified mutations in the *GRIN2A* gene in patients with different forms of epilepsy, including early-onset epileptic encephalopathy, continuous spike-and-waves during slow-wave sleep syndrome (CSWSS), Landau-Kleffner syndrome (LKS), and Rolandic epilepsy [13,15,18,21–24].

In this study, next generation sequencing identified a *GRIN2A* missense mutation c.2191G>A (p.Asp731Asn, hereafter referred to as GluN2A-D731N) from a pediatric patient diagnosed with epilepsy and DD. The patient’s clinical features were summarized and compared with the two previously reported patients with the same mutation. The influence of the mutation on NMDA receptor function was evaluated here electrophysiologically *in vitro* by using two-electrode voltage clamp current recordings and whole cell voltage clamp current recordings.

## Materials and methods

### Ethics statement

Written informed consent was obtained from the parents of all the patients. This study was approved by the Peking University First Hospital Medical Ethics Committee. All data of this study were analyzed anonymously.

### Patient’s information

The patient with *GRIN2A* mutation was collected from the Department of Pediatrics, Peking University First Hospital in 2013. This patient was clinically diagnosed as having epilepsy and ID/DD of unknown origin. Nevertheless, it was strongly suspected that the etiology of these patients’ diseases was genetic, as there was: (1) no definite perinatal brain injury; (2) no hypoxia, ischemia, infection of the central nervous system or cranial trauma; (3) no evidence of typical inherited metabolic disorders or specific neurodegenerative disorders based on clinical features, neuroimaging or blood/urinary metabolic diseases screening; (4) normal routine karyotyping.

### Genetic analysis

We constructed a custom-designed panel capturing the coding exons of 300 genes associated with epilepsy and IDDs, including *GRIN2A* [25]. This panel was synthesized using the Agilent SureSelect Target Enrichment technique, containing a total of 11,417 probes covering 1.285 Mbp. Targeted next generation sequencing (NGS) was subsequently performed on an Illumina GAIIx platform (Illumina, San Diego, CA, USA) using a paired-end sequencing of 110 bp to screen for mutations. Image analysis and base calling were performed by RTA software (real-time analysis, Illumina) and CASAVA software v1.8.2 (Illumina). After marking duplicate reads and filtering out reads of low base quality score using the Genome Analysis Tool kit (GATK), clean paired-end reads were aligned to GRCh27/hg19 using BWA software (Pittsburgh Supercomputing Center, Pittsburgh, PA, USA). In addition to insertion-deletions (indels) and single-nucleotide polymorphisms (SNPs) identified using the GATK, variants were annotated using ANNOVAR. At last, we performed validation and parental origin analyses for the mutation by conventional Sanger sequencing.

### Site-directed mutagenesis, RNA synthesis, and injection

All *in vitro* studies were conducted according to the guidelines of Emory University. Mutagenesis was performed using the *QuikChange* protocol with *Pfu* DNA polymerase (Stratagene La Jolla, CA, USA) to replicate the parental DNA strand with the desired mismatch incorporated into the primer. Methylated parental DNA was digested with Dpn I for 3 hours at 37°C and the nicked mutant DNA was transformed into TOP10 Competent Cells (Life Tech, Grand Islands, NY, USA). Bacteria were spun down and prepared using the Qiagen Qiaprep Spin Miniprep kit (Hilden, Germany). Sequences were verified through the mutated region using dideoxy DNA sequencing (Eurofins MWG Operon, Huntsville, AL, USA). The plasmid vector for wild type (WT) human GluN1-1a (hereafter GluN1) and GluN2A was pCI-neo [22] (GenBank accession codes: NP_015566, NP_00082).

To investigate the effect of a single copy mutant on the channel function, the constructs of tri-heteromeric receptors were generated using rat GluN1-1a (hereafter GluN1) and GluN2A with modified C-terminal peptide tags. As described by Hansen et al. [26], two peptide tags (C1 and C2) were generated and allowed to form a coiled-coil interaction that masked the dilycine KKTN retention motif. These C-terminal retention signals were fused to the wild type and mutant GluN2A receptors to generate GluN2A-C1, GluN2A-C2, GluN2A-C1-D731N, GluN2A-C2- D731N. Only receptors with one copy of a C1 tag and one copy of a C2 tag will mask the endoplasmic reticulum retention signal and reach the cell membrane surface. Co-expressing with rat GluN1 with C1- and C2-tagged GluN2A yielded receptors with the following subunit combinations: GluN1/GluN2A-C1/GluN2A-C2 (referred to 2A/2A), GluN1/GluN2A-C1-D731N/GluN2A-C2 (referred to D731N/2A), and GluN1/GluN2A-C1-D731N/GluN2A-C2-D731N (referred to D731N/D731N).

Complementary DNA (cDNA) was linearized using FastDigest (Thermo, Waltham, MA, USA) restriction digestion at 37°C for 1 hour. Complementary RNA (cRNA) was made from linearized cDNA for wild type and mutants using the mMessage mMachine T7 kit (Ambion, Austin, TX, USA). Injections of cRNA into *Xenopus Laevis* oocytes (Ecocyte, Austin, TX, USA) was performed as previously described [27]. Oocytes were held in Barth’s solution containing (in mM) 88 NaCl, 2.4 NaHCO_3_, 1 KCl, 0.33 Ca(NO_3_)_2_, 0.41 CaCl_2_, 0.82 MgSO_4_, 5 Tris/HCl (pH 7.4 with NaOH) supplemented with 100 μg/mL gentamycin, 40 μg/mL streptomycin, and 50 μg/mL penicillin at 15–19°C. Injection of cRNA was done in a 1:2 GluN1:GluN2A ratio for the di-heteromeric receptors and 1:1:1 GluN1:GluN2A-C1:GluN2A-C2 ratio for the tri-heteromeric receptors by weight with injections totaling 5–10 ng of cRNA in water, with 50 nL injected per oocyte. Control experiments evaluating the escape of non-triheteromeric receptors from the ER retention were performed [26]. This evaluation estimated that <3% of the current responses in the tri-heteromeric experiments were mediated by non-triheteromeric receptors.

### Two-Electrode Voltage Clamp Current (TEVC) recordings

Voltage clamp recordings were performed 2–4 days post-injection at room temperature (23°C). The extracellular recording solution contained (in mM) 90 NaCl, 1 KCl, 10 HEPES, 0.5 BaCl_2_, and 0.01 EDTA (pH 7.4 with NaOH). Solution exchange was computer controlled through an 8-valve positioner (Digital MVP Valve, Hamilton, CT, USA). Oocytes were placed in a dual track chamber that shared a single perfusion line, allowing simultaneous recording from two oocytes. All concentration-response solutions were made in the extracellular recording solution. Voltage control and data acquisition were performed with a two-electrode voltage-clamp amplifier (OC725, Warner Instruments, Hamden, CT, USA). The voltage electrode was filled with 0.3 M KCl and the current electrode with 3 M KCl. Oocytes were held under voltage clamp at -40 mV unless otherwise indicated. For the experiments with glutamate concentration ≥ 3 mM, osmolality was compensated for solutions with high concentrations of glutamate by adding sodium isethionate; the pH was corrected after addition of glutamate. Experiments assessing inhibition by extracellular Zn^2+^ were performed in the presence of tricine (10 mM) at pH 7.3 with voltage held at -20 mV. ZnCl_2_ solutions (10 mM) were made fresh daily in deionized nuclease-free water and added directly to recording solution to obtain the desired nominal Zn^2+^ concentration^25^. Experiments assessing the sensitivity of the channel to Mg^2+^ blockade were performed at a holding potential of -60 mV. The effects of Mg^2+^, proton, zinc, (+)MK801 maleate (R&D Systems, Inc., Minneapolis, MN, USA), and MTSEA (2-aminoethyl methanethiosulfonate hydrobromide, Toronto Research Chemicals, Ontario, Canada) were evaluated on the current response to 4.0–30,000 μM glutamate and 100 μM glycine. For the experiments of MK801 and MTSEA, the EC_50_ concentrations of glutamate were used to activate corresponding receptors. All chemicals were from Sigma-Aldrich unless otherwise stated.

### Whole-cell voltage-clamp recordings from transfected HEK cells

HEK293 cells (CRL 1573, ATCC, Manasas, VA, USA) were plated on glass coverslips coated with 0.1 mg/ml poly-D-lysine and cultured at 37°C in standard media (5% CO_2_ in DMEM/GlutaMax with 10% fetal bovine serum and 10 U/ml penicillin-streptomycin). The calcium phosphate method [28] was used to co-transfect HEK293 cells with cDNA encoding GFP, GluN1, and GluN2A or GluN2A(D731N). After 24 hr, whole-cell voltage clamp current recordings were performed at 23°C at a holding potential of -60 mV using an Axopatch 200B amplifier (Molecular Devices, Sunnyvale, CA, USA) in recording solution containing (in mM) 150 NaCl, 10 HEPES, 30 D-mannitol, 3 KCl, 1.0 CaCl_2_, and 0.01 EDTA (pH 7.4). Recordings were made by recording electrodes (3–5 MΩ) filled with (in mM) 110 D-gluconate, 110 CsOH, 30 CsCl, 5 HEPES, 4 NaCl, 0.5 CaCl_2_, 2 MgCl_2_, 5 BAPTA, 2 NaATP, and 0.3 NaGTP (pH 7.35). Rapid solution exchange was achieved by using a two-barrel theta glass pipette controlled by a piezoelectric translator (Siskiyou Corporation, Grants Pass, OR, USA). Open-tip solution exchange time was < 1 ms. The data were acquired using Clampex 10 (Molecular Devices, Sunnyvale, CA, USA). Time constants describing the deactivation time course were determined using ChanneLab (Synaptosoft, Decatur, GA, USA) to fit a two-component exponential function to the current response time course following glutamate removal,

$$Amplitude(t)={Amp}_{FAST}\times exp(-time/\tau_{FAST})+{Amp}_{SLOW}\times exp(-time/\tau_{SLOW})$$ Equation-1

where *Amp*_*FAST*_ and *Amp*_*SLOW*_ are the amplitude of the fast and slow components.

## Data and statistical analysis

The EC_50_ value is the concentration of agonist that elicits a half maximal excitatory response, and was determined by

$$Response=100\%/\left(1+{\left({EC}_{50}/\left[agonist\right]\right)}^{nH}\right)$$ Equation-2

where *nH* is the Hill slope of the response curve. The IC_50_ value is the concentration of antagonist that elicits a half-maximal inhibitory response, and was calculated using

$$Response=\left(100\%-minimum\right)/\left(1+{\left(\left[concentration\right]/{IC}_{50}\right)}^{nH}\right)+minimum$$ Equation-3

where *minimum* is the residual response at saturating concentrations of inhibitor (Mg^2+^, protons, or Zn^2+^).

The channel open probability (*P*_*OPEN*_) of the mutant receptors was assessed using the kinetics of MK801 inhibition by TEVC recordings. The onset of MK801 inhibition was fitted to a single exponential function (ChanneLab, Synaptosoft, Decatur, GA, USA) to determine *tau*_*on*_ according to

$$Amplitude(t)=Amplitude\times exp(-time/{tau}_{on})$$ Equation-4

*P*_*OPEN*_ was estimated from

$$P_{OPEN}(mutant)=P_{OPEN}(WT)\times k_{on}(mutant)/k_{on}(WT)$$ Equation-5

Assuming that the binding of MK801 is irreversible over the time course of the experiment, the microscopic association rate for MK801, *k*_*on*_, is 1/(*tau*_*on*_ *×* [*concentration*]), where *concentration* was 0.2 μM, and P_OPEN_ for WT receptors was taken to be 0.278 for di-heteromeric receptors (WT 2A; calculated P_OPEN_: 0.278 ± 0.017 from 252 ± 16% of MTSEA potentiation by Equation-6 below, n = 13) and 0.370 for tri-heteromeric receptors (WT 2A/2A; Supplemental Fig 2 in [29]) in the presence of saturating concentrations of agonists (≥ 100 μM for both glutamate and glycine)

The channel open probability was also evaluated and calculated by the degree of MTSEA potentiation on the currents evoked by EC_50_ concentrations of glutamate and 100 μM glycine by using [30,31]:

$$P_{OPEN}=(\gamma_{MTSEA}/\gamma_{CONTROL})\times (1/Potentiation)$$ Equation-6

where *Potentiation* is the current after MTSEA treatment divided by the current before treatment and γ is the chord conductance measured before and after MTSEA treatment.

Statistical significance was computed using unpaired *t* test (two-tailed) or one way ANOVA with *post hoc* Tukey test, with p < 0.05 considered significant. Data are presented as mean ± standard error of the mean (SEM). Error bars represent SEM unless otherwise stated.

## Results

### Identification of GluN2A(D731N) mutation

**Fig 1:**
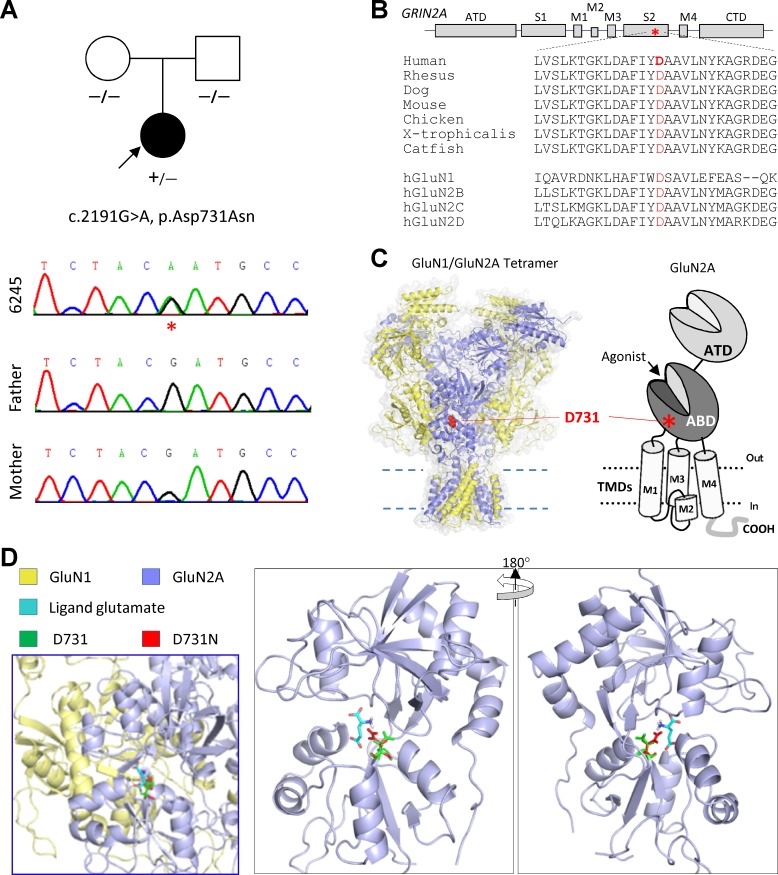
Genetic and protein changes of *GRIN2A* and GluN2A. (**A**) Family pedigree and genotypes (indicated by *) reveal a *de novo* mutation (affected proband is indicated by arrow; parentage was confirmed by Sanger sequencing). (**B**) Schematic representation of GluN2A subunit (asterisk indicates the position of the D731N mutation). The residue aspartic acid at position 731 is highly conserved across vertebrate species, and other GluN subunits. (**C**) A homology model of GluN1/GluN2A complex built from the GluN2B crystallographic data [32,33] with Asp731 shown as spacefill in red. The red asterisk in the cartoon illustrating the domain arrangement of an individual GluN subunit (right panel) shows the position of Asp731 in the agonist-binding domain (S2, ABD). Panel **D** shows glutamate binding pocket depicting the position of D731 (in GREEN) and D731N (in RED) in the GluN2A ABD structure in complex with ligand glutamate (in CYAN).

We identified a *de novo* missense mutation c.2191G>A (p.Asp731Asn, D731N; Fig 1A) in a patient with focal epilepsy and acquired epileptic aphasia, a heterozygous *GRIN2A* mutation in a portion of the gene that is intolerant to change. The result is an aspartic acid to asparagine missense mutation at residue 731 in the extracellular agonist-binding domain (ABD) of the GluN2A subunit. This area is highly conserved across different vertebrate species as well as among all of the other NMDAR subunits (Fig 1B), indicating a possible important role in NMDAR function. The segment of the polypeptide chain harboring residue 731, often referred to as the S2 region, largely forms membrane-proximal half of the bi-lobed clamshell responsible for binding agonist, and is composed of the polypeptide chain between the M3 and M4 transmembrane domains (Fig 1C). The residue Asp at position 731 resides within the glutamate binding pocket (Fig 1D), and the substitution of Asn at this position may interfere glutamate ligand binding.

### Patient information

**Fig 2:**
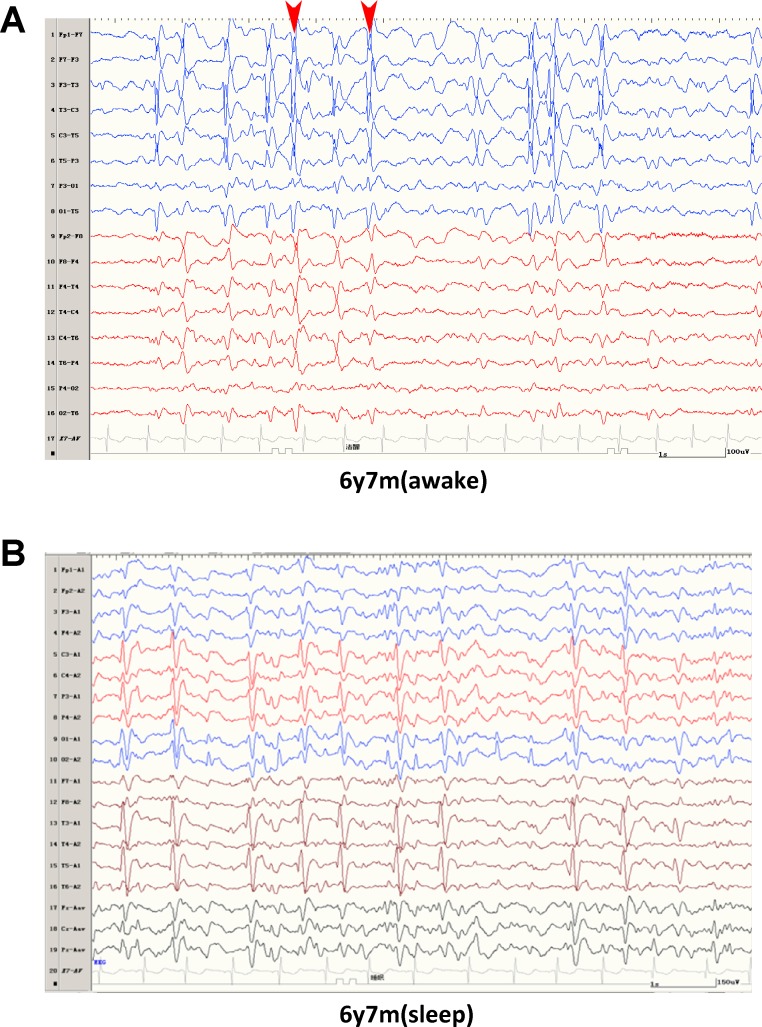
EEG of patient 6245 at 6y7m. (A) During awake period, spike and spike-wave complexes (arrow heads) in Rolandic region, more obvious on the left side (blue). (B) During sleep period, NREM (non-rapid eye movement) index was about 85%.

The patient was an 11-year-old girl with partial seizures that started at four years of age, with seizures restricted to periods of sleep. Until now, she has been treated with four antiepileptic drugs (AEDs), including valproate and oxcarbazepine, which had no obvious effect. In addition, levetiracetam and clonazepam partially controlled the seizures. She manifested developmental delay from one-year-old and started motor and cognitive function regression, especially verbal dyspraxia at four-year-old after seizures happened. She also suffered from paroxysmal weakness of her right lower limb and gait abnormality. Her prenatal history was normal and neurological examination was unremarkable. Her electroencephalograph (EEG) recordings (at 6 years) showed that background activities were slow, and that spike and spike-wave complexes were present in bilateral rolandic regions, with a significant increase during sleep; discharge index was about 85% in non-rapid eye movement (NREM) sleep (Fig 2). Her cranial magnetic resonance imaging (MRI) was normal (data not shown). Two additional patients with the same missense mutation have separately been reported [15,34], however no functional analysis on the effects of this mutation were performed on human NMDARs. Thus, a total of three unrelated non-familial patients that have been identified with the same missense mutation and a similar, if not identical, presentation of symptoms including seizures, cognitive deficits, and motor deficits (Table 1).

**Table 1:** Summary of patients' information.

	Patient 1	Patient 2	Patient 3
**Mutation**	*De novo* GRIN2A c.2191G>A (p.Asp731Asn)	Inherited GRIN2A c.2191G>A (p.Asp731Asn)	*De novo* GRIN2A c.2191G>A (p.Asp731Asn)
**Family History**	None	Same mutation in mother only had VD	Same mutation in daughter
**Diagnoses**	aRE, VD	aRE, VD	LKS
**Seizure Type**	PS	GTCS	PS, GTCS (resolved in late childhood)
**Onset Age**	4 yrs	2 yrs	5 yrs
**ID/DD**	Motor and cognitive developmental delay, VD	Intellectual, motor and cognitive regression, Psychomotor and language delay	N/A
**EEG**	Discharges in rolandic region, ESES	CTS	Sharp slow-wave complexes in temporal lobes
**MRI/CT**	normal	N/A	normal
**Source**	this study	Lesca et al., Nat Genet 2013	Dyment et al., Clin Genet 2015

aRE: atypical Rolandic Epilepsy; CTS: cerebrotemporal spikes; ESES: Electrical Status Epilepticus in sleep; GTCS: General tonic-clonic seizures; LKS: Landau-Kleffner Syndrome; N/A: not available; PS: partial seizures; VD: verbal dyspraxia.

### GluN2A(D731N) mutation changes agonist glutamate potency

**Fig 3:**
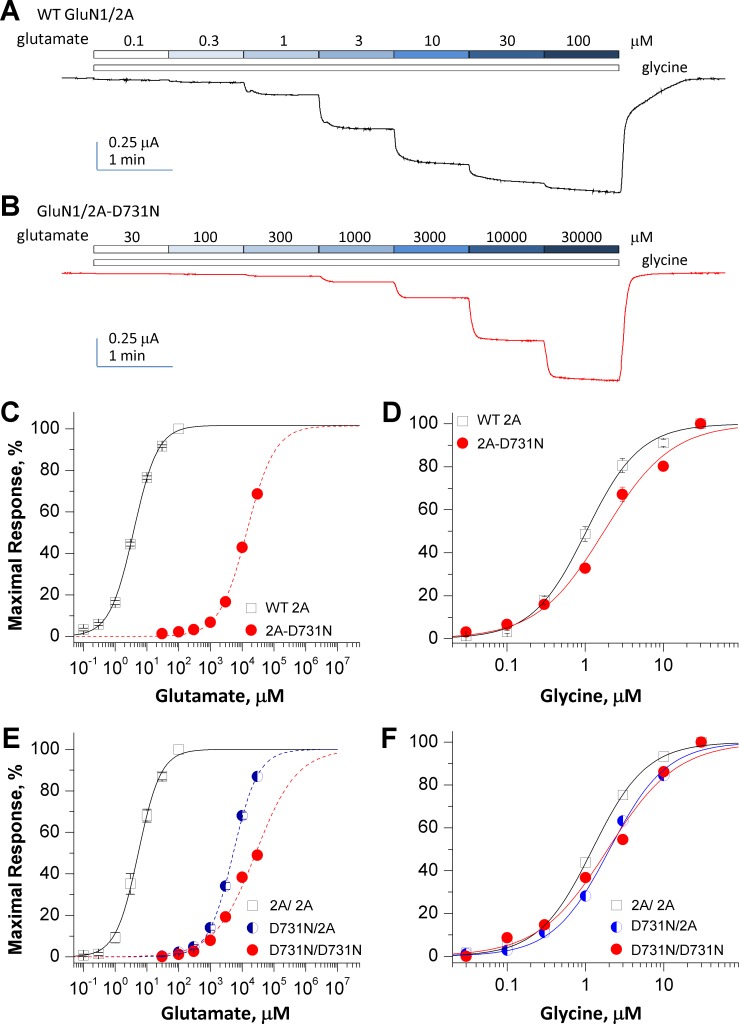
GluN2A(D731N) reduces the agonist potency. (**A,B**) Representative TEVC recordings obtained from oocytes expressing WT GluN1/GluN2A (WT 2A) receptors (**A**) and GluN1/GluN2A(D731N) (2A-D731N) receptors (**B**) in which the currents were evoked by increasing concentrations (μM) of glutamate (in the presence of 100 μM glycine) at the holding potential of -40 mV. (**C,D**) Composite concentration-response curves of glutamate and glycine for di-heteromeric receptors GluN1/GluN2A (WT 2A) and GluN1/GluN2A-D731N (2A-D731N). (**E,F**) Composite concentration-response curves of glutamate and glycine for tri-heteromeric receptors GluN1/GluN2A/GluN2A (2A/2A), GluN1/GluN2A(D731N)/GluN2A (D731N/2A) and GluN1/GluN2A(D731N)/GluN2A(D731N) (D731N/D731N). (**C,E**) The composite glutamate (in the presence of 100 μM glycine) concentration-response curves reveal a significant decrease in glutamate potency in both di-heteromeric (**C**) and tri-heteromeric (**E**) GluN2A(D731N)-containing NMDARs compared to wild type receptors. A single copy D731N-containing receptor (D731N/2A) (**E**) showed an intermediate but a dominantly negative effect on glutamate potency. The traces for D731N-contianing receptors (dash lines in panels **C** and **E**) were fitted by predicted glutamate concentrations of maximal responses. (**D,F**) The composite glycine (in the presence of 3–30 mM glutamate) concentration-response curves indicate a mild, but significantly reduced glycine potency in both di-heteromeric (**D**) and tri-heteromeric (**F**) GluN2A(D731N) receptors. Error bars are SEM, and are shown when larger than symbol size.

Two-electrode voltage clamp current recordings from *Xenopus laevis* oocytes (Fig 3A and 3B**)** were performed to evaluate the effect of the GluN2A mutation on NMDAR function. Concentration-response curves (Fig 3) were generated for the endogenous NMDAR co-agonists glutamate and glycine to evaluate whether the mutation changes agonist potency. The analyses of these data show that the GluN2A(D731N)-containing NMDA receptors had a significantly lower glutamate potency, with the EC_50_ value being increased from 3.7 μM in WT GluN2A receptors to about 13.7 mM in GluN2A(D731N)-containing NMDA receptors (Fig 3C; Table 2) when the concentration-response curves were fitted (dash lines in Fig 3) with predicted maximal response since the saturating glutamate concentrations are unknown. There is also a modest, but significant, decrease in glycine potency from an EC_50_ value of 1.0 μM in WT NMDAR receptors to 1.7 μM in GluN2A (D731N)-containing NMDARs (Fig 3C and 3D; Table 2). These data suggest that GluN2A(D731N) mutation decreases the potency of glutamate by over 3,000-fold, suggesting high concentrations of glutamate are needed in the brain to activate the NMDARs harboring this mutation.

**Table 2:** Summary of pharmacological data for GluN2A(D731N).

	di-heteromeric receptors		tri-heteromeric receptors		
	WT 2A	2A-D731N	WT 2A/2A	D731N/2A	D731N/D731N
**Glutamate, EC**_**50**_ **(n)**	3.7 ± 0.1 (7)	13,655 ± 199 (11) ^¥^*	5.7 ± 0.5 (10)	6,451 ± 260 (16) ^¥^ ^#^	30,469 ± 438 (11) ^¥^ ^#^ ^%^
**Glycine, EC**_**50**_ **(n)**	1.1 ± 0.1 (10)	1.7 ± 0.2 (10)*	1.3 ± 0.1 (8)	2.1 ± 0.1 (12) ^#^	2.0 ± 0.2 (8) ^#^
**Proton, IC**_**50**_	6.8 (5)	7.3 (6)	—	—	—
**Proton, % (n)**^**£**^	54 ± 1.4 (10)	34 ± 1.7 (8)*	59 ± 1.8 (11)	37 ± 1.8 (8) ^#^	33 ± 3.9 (8) ^#^
**Zinc, IC**_**50**_ **(n)**	87 ± 12 (5)	20 ± 5.6 (8)*	—	—	—
**Zinc, max% (n)**^**$**^	36 ± 1.5 (5)	58 ± 2.2 (8)*	—	—	—
**Mg**^**2+**^**, IC**_**50**_ **(n)**^**&**^	31 ± 4.1 (20)	25 ± 4.1 (15)	—	—	—

The data were generated by TEVC recordings on *Xenopus* oocytes and were expressed as mean ± s.e.m. (n) is the number of cells recorded from.

^¥^ fitted EC_50_ values by predicted glutamate concentrations of maximal responses

* p < 0.01, compared with WT 2A, unpaired t-test

^#^ p < 0.01 compared with WT 2A/2A

^%^ p < 0.01 compared with D731N/2A, one way ANOVA, *post hoc* Tukey test

^£^ percentage current at pH 6.8 compared to the pH 7.6

^$^ maximal inhibition at 300 nM Zn^2+^

^&^ holding at -60 mV

Since the mutation in these patients is heterozygous and the functional NMDAR complex contains two copies of GluN2 subunit, we expect some NMDARs in these individuals may have a single copy of mutant GluN2A(D731N). We therefore engineered a pair of modified GluN2A subunits that contain complementary sets of coiled-coil domains followed by an endoplasmic reticulum retention signal to control receptor trafficking and subunit composition on the cell surface [26,34]. We generated receptors containing 0, 1 and 2 copies of the GluN2A(D731N) on the cell surface and repeated the experiments that establish the concentration-effect relationship to investigate the effects of a single copy of GluN2A(D731N) on agonist potency. The data showed that a single copy of GluN2A(D731N) produced an intermediate, but strong decrease in glutamate potency (2A/2A: 5.7 ± 0.5 μM; D731N /2A: 6,451 ± 260 μM; D731N/D731N: 30,469 ± 438 μM) (Fig 3F; Table 2). Again, the concentration-response curves were fitted (dash lines in Fig 3F) with predicted maximal response since the saturating glutamate concentrations could not be determined. Similar to the di-heteromeric mutant receptors (2A-D731N), NMDARs that contained one or two copies of GluN2A(D731N) showed a mild, but significant reduction in glycine potency (2A/2A: 1.3 ± 0.1 μM; D731N /2A: 2.1 ± 0.1 μM; D731N/D731N: 2.0 ± 0.2 μM) (Fig 3F; Table 2). These results confirm that a single copy of GluN2A(D731N)-containing NMDARs can significantly alter receptor function.

### GluN2A(D731N) mutation changes sensitivity to negative allosteric modulators

Negative regulation by endogenous extracellular magnesium, zinc and protons is an important feature of NMDAR function [1]. The sensitivity of the mutant receptors to extracellular Mg^2+^, Zn^2+^, and H^+^ was evaluated by generating concentration-response curves using two electrode voltage clamp current recordings to calculate the concentration required to produce half maximal inhibition of responses (IC_50_).

**Fig 4:**
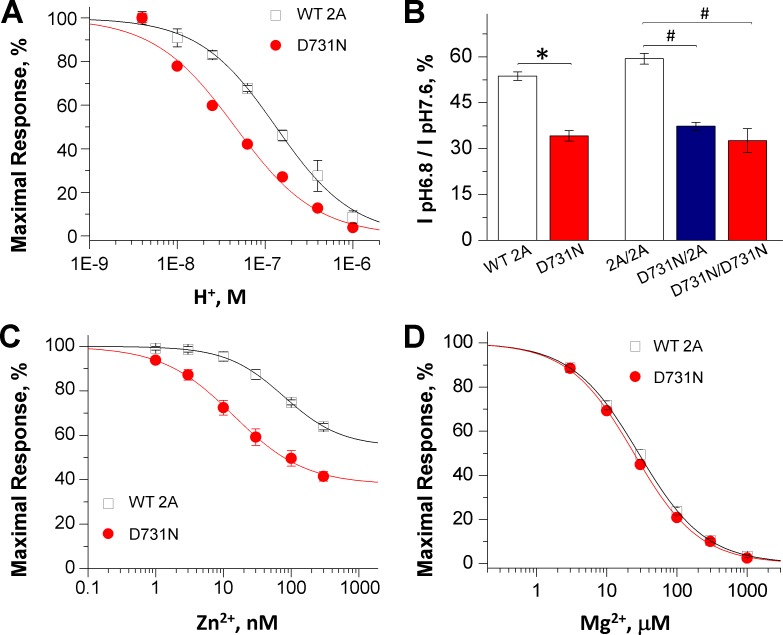
GluN2A(D731N) enhances sensitivity to endogenous proton and zinc ions. (**A**) The composite proton concentration-response curves show an enhanced sensitivity of the GluN2A(D731N)-containing receptors to proton compared to the WT NMDA receptors; the abscissa shows hydrogen ion activity. (**B**) Summary of proton sensitivity of WT GluN2A and mutant receptors, evaluated by current ratio at pH 6.8 to pH 7.6 (in the presence of 30 mM glutamate and 100 μM glycine). Di-heteromeric (h2A-D731N), one-copy and two-copy mutant tri-heteromeric (D731N/2A and D731N/D731N) receptors show a decreased current ratio, indicating enhanced proton sensitivity. (**C**) The composite zinc concentration-response curves show an enhanced sensitivity of the GluN2A(D731N)-containing receptors to zinc compared to the WT NMDA receptors. (**D**) Composite Mg^2+^ concentration-response curves for di-heteromeric receptors indicate a similar Mg^2+^ inhibition of GluN2A(D731N)-containing receptors. The data were generated by TEVC recordings on *Xenopus* oocyte at holding potential of -40 mV for proton concentration-response curves, and -20 mV for zinc, and -60 mV for Mg^2+^ concentration-response curves. Fitted parameters are in Table 2.

Analysis revealed that GluN2A(D731N)-containing receptors significantly increase the proton sensitivity as measured by IC_50_ corresponding to pH 7.3 for the mutant, compared to pH 6.8 of WT GluN2A (**Fig 4A; Table 2**). The di-heteromeric mutant receptors showed significantly less current remaining at pH 6.8 compared to pH 7.6 (34%) than the WT receptors (54%; **Fig 4B; Table 2**), indicating an enhanced proton inhibition on the mutant receptors. One-copy and two-copy D731N-containing NMDARs also showed significantly less current remaining at pH 6.8 compared to pH 7.6 than the WT receptors (2A/2A: 59%, D731N/2A: 37%, and D731N/2A: 33%). In addition, the GluN2A(D731N)-containing receptors showed an increased degree of maximal inhibition by 300 nM Zn^2+^, which was 58% in GluN2A(D731N) compared to 36% for the WT receptors (Fig 4C; Table 2). Mg^2+^ potency was not significantly affected by GluN2A(D731N) (Fig 4D; Table 2). Altogether, these results suggest that lower concentrations of negative allosteric modulators Zn^2+^ and H^+^ can inhibit the mutant NMDAR receptors harboring D731N compared to the WT receptors, further contributing to NMDAR hypofunction.

### GluN2A(D731N) mutation changes synaptic-like response time course

**Fig 5:**
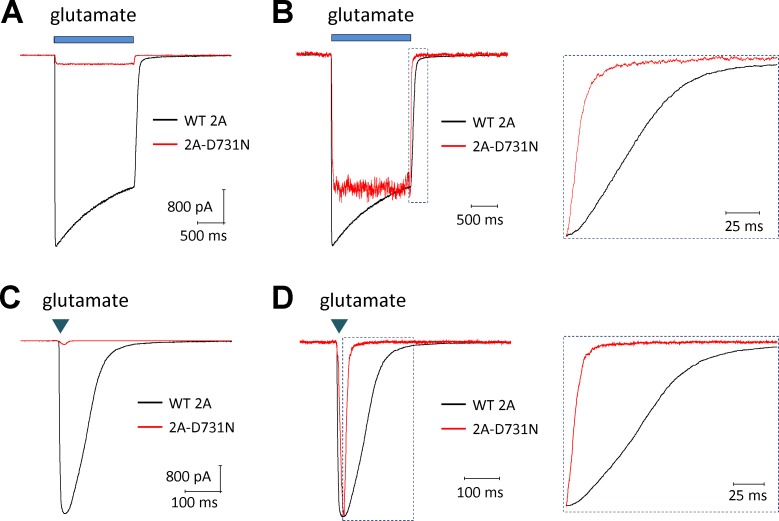
GluN2A(D731N) decreases current amplitudes and shortens synaptic-like response time course. The representative current response time course was generated by the whole cell voltage clamp recording (V_HOLD_ -60 mV) of GluN1/GluN2A (WT 2A, in BLACK) and GluN1/GluN2A-D731N (2A-D731N, in RED) receptor responses to rapid application of long (1.5 sec) (**A,B**) and brief (5 ms) (**C,D**) application of 30 mM glutamate. Panels **B** and **D** showed normalized responses to the WT response at the moment glutamate were removed. The mutant D731N-containing receptors showed an accelerated deactivation time course (*right panel* in **B,D**). Saturating glycine (100 μM) was present in all of solutions. Fitted parameters describing the response time course are given in **Table 3.**

The response time course following rapid removal of glutamate from NMDARs has been suggested to control the time-course of the NMDAR component of the EPSC (excitatory postsynaptic current) [35]. To evaluate the effects of GluN2A(D731N) on the deactivation time course, we measured current responses following glutamate removal using a rapid solution exchange system in whole cell voltage clamp current recordings from transiently transfected HEK293 cells expressing WT GluN1/GluN2A or GluN1/GluN2A(D731N). GluN2A(D731N) significantly reduced amplitude of current response to 1.5 sec application (prolonged application) of 30 mM glutamate in the presence of 100 μM glycine (5.1 pA/pF vs. 235 pA/pF of WT; Fig 5A; Table 3), which is consistent with the effect of this mutation on receptor surface trafficking [20]. The mutant receptors indicated a shortened glutamate deactivation time course fitted by two exponential components, with a weighted τ_w_ of 18 ms compared to 72 ms for WT GluN1/GluN2A (p < 0.01, unpaired t-test; Fig 5B; Table 3). Charge transfer during synaptic transmission can be determined from the integral of experimentally recorded EPSCs, which can be approximated by an instantaneously rising and exponentially decaying function. The integral of this function is the product of the amplitude and the weighted time constant describing the exponential decay. We estimate the synaptic charge transfer by using the product of the response amplitude and deactivation time courses, which was markedly decreased by over 180-fold for GluN2A(D731N) compared to wild type GluN2A (Table 3). To mimic synaptic events, we also measured current responses by briefly moving the cell into the agonist solution for 5 milliseconds (brief application). Similar to the prolonged (1.5 sec) application of glutamate, GluN2A(D731N) had a faster deactivation time course with a τ_W_ of 13 ± 2.0 ms compared to 55 ± 8.4 ms for WT GluN2A (p < 0.01, unpaired t-test; Fig 5C and 5D). These data suggest NMDARs that contained GluN2A(D731N) have a shortened deactivation response time course, and thus a shortened time course of the NMDAR component of the EPSC at synapses.

**Table 3:** Summary of response time course for GluN2A(D731N).

	WT 2A	2A-D731N
**Amplitude (peak, pA/pF)**	235 ± 46	5.1 ± 1.1 *
**Amplitude (SS, pA/pF)**	126 ± 28	4.8 ± 1.0 *
**ISS / IPEAK**	0.56 ± 0.01	0.94 ± 0.01 *
**10–90% Rise time (ms)**	4.9 ± 0.4	17 ± 2.2 *
τ_**FAST**_ **deactivation (ms)**	41 ± 3.7	15 ± 2.4 *
τ_**SLOW**_ **deactivation (ms)**	384 ± 96	37 ± 12 *
**%**τ_**FAST**_ **deactivation**	81 ± 6.5	74 ± 6.2 *
τ_**W**_ **deactivation (ms)**^**$**^	72 ± 9.2	18 ± 2.3 *
**Charge transfer, pA x ms/pF**	16,984	92 *
**n**	12	11

The data were generated by whole cell voltage clamp current recordings on transfected HEK293 cells and were expressed as mean ± s.e.m.

^$^ The weighted tau was calculated from the Amplitude of the fast and slow components (*Amp*_*FAST*_, *Amp*_*SLOW*_) by τ_**W**_ = *Amp*_*FAST*_/(*Amp*_*FAST*_+*Amp*_*SLOW*_) *×* τ_**FAST**_ **+** *Amp*_*SLOW*_/(*Amp*_*FAST*_+*Amp*_*SLOW*_) *×* τ_**SLOW**_

* p < 0.01, compared with WT 2A, unpaired t-test

### GluN2A(D731N) mutation changes channel open probability

**Fig 6:**
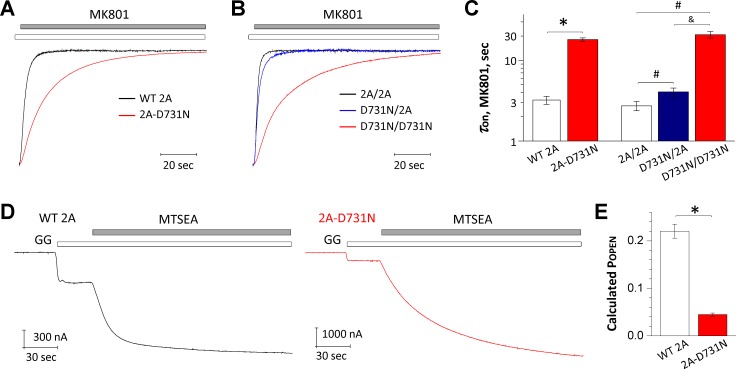
GluN2A(D731N) decreases channel open probability. (**A-C**) Representative TEVC recordings from WT and the mutant di-heteromeric and tri-heteromeric receptors from *Xenopus* oocytes show the time course of MK801 (0.2 μM) inhibition. The receptors were activated by EC_50_ concentrations of glutamate with saturating concentration (100 μM) of glycine at holding potential of -40 mV. The di-heteromeric mutant (2A-D731N) and tri-heteromeric receptors with two-copies of the mutant subunit (D731N/D731N) showed a prolonged inhibition rate, reflecting a decreased channel open probability. A single copy mutant subunit (D731N/2A) also produced a mild but significant prolongation in MK801 inhibition rate. (**D,E**) The channel open probability was evaluated by measuring the degree of MTSEA (200 μM) potentiation using TEVC recordings from *Xenopus* oocytes expressing the WT GluN2A (*left panel*) or the mutant GluN2A(D731N) (*right panel*) coexpressed with GluN1(A652C) at a holding potential of -40 mV in presence of EC_50_ concentrations of glutamate with saturating concentration (100 μM) of glycine (open bar) and 0.2 mM MTSEA (closed bar). Fitted parameters describing the exponential time course for MK801 inhibition and calculated open probability are given in Table 4.

To evaluate the effects of this mutant on channel open probability, the receptors were activated by EC_50_ concentrations of glutamate (4 μM for WT 2A, 13.7 mM for 2A-D731N, 6 μM for 2A/2A, 6.5 mM for D731N/2A, and 30 mM for D731N/D731N, see Table 2) with saturating concentration (100 μM) of glycine. The rate of channel block by 200 nM MK801 was measured using TEVC recordings from *Xenopus* oocytes to estimate channel open probability [28,36,37]. The di-heteromeric mutant (2A-D731N) receptors show a slower rate of inhibition, which we interpret as a decreased channel open probability. We estimate the reduction in open probability to be 6.0-fold (0.046 compared to 0.28 of WT 2A; Fig 6A and 6C; Table 4; see Methods). In NMDARs that contain two copies of the mutation (D731N/D731N), there was a 6.1-fold reduction in open probability (0.064) compared to the 0.37 of WT 2A/2A. NMDARs with a single copy of the mutation (D731N/2A) showed 1.5-fold decrease in open probability (0.249) (Fig 6B and 6C; Table 4).

**Table 4:** Summary of channel open probability for GluN2A(D731N).

	di-heteromeric receptors		tri-heteromeric receptors		
	WT 2A	2A-D731N	WT 2A/2A	D731N/2A	D731N/D731N
***1/Tau*-MK801, ms**^**-1**^	0.334 ± 0.026	0.056 ± 0.002 *	0.405 ± 0.04	0.273 ± 0.035 ^#^	0.066 ± 0.016 ^#^ ^%^
**POPEN (from MK801)**	0.278 ± 0.024	0.046 ± 0.002 *	0.370 ± 0.039	0.249 ± 0.032 ^#^	0.064 ± 0.014 ^#^ ^%^
**n**	8	10	8	8	17
**MTSEA Potentiation (% of control)**	312 ± 17	1541 ± 106 *	—	—	—
**POPEN (from MTSEA)**	0.222 ± 0.015	0.047 ± 0.003 *	—	—	—
**n**	10	16	—	—	—

Evaluated by TEVC recordings on *Xenopus* oocytes, see Materials and Methods.

The data were expressed as mean ± s.e.m. (n) is the number of cells recorded from.

* p < 0.01, compared with WT 2A, unpaired t-test

^#^ p < 0.01 compared with WT 2A/2A

^%^ p < 0.01 compared with D731N/2A, one way ANOVA, *post hoc* Tukey test

We further evaluated channel open probability by measuring the degree of MTSEA (200 μM, closed bar in Fig 6D) potentiation using TEVC recordings from *Xenopus* oocytes expressing the WT GluN2A (*left panel*, Fig 6D) or the mutant GluN2(D731N) (*right panel*, Fig 6D) coexpressed with GluN1(A652C) at holding potential of -40 mV. Because the low potency of glutamate at mutant receptors prevented us from using saturating concentrations of glutamate, we assessed MTSEA potentiation on responses activated by EC_50_ concentrations of glutamate (4.0 μM glutamate for WT GluN2A and 13.7 mM glutamate for GluN2A(D731N) with 100 μM glycine. Channel open probability is inversely correlated with the degree of potentiation [30], and thus we interpret the increase in the current response produced by MTSEA treatment of GluN1(A652C)/GluN2A(D731N) receptors to reflect a significant decrease in open probability by the D731N mutation (calculated P_OPEN_ 0.05 for 2A-D731N *vs*. 0.22 for WT 2A; Fig 6E; Table 4). These data are consistent with the change in the time course of the onset of MK801 inhibition, indicating the mutant D731N receptors decrease channel open probability.

## Discussion

Recent advances in whole exome sequencing have improved affordability of genotyping and aided in understanding undiagnosed diseases as well as epilepsy syndromes. This has led to compelling data that NMDAR mutations contribute to epilepsy with studies suggesting that *GRIN2A* mutations are associated with 9% of epilepsy-aphasia spectrum disorders and 20% of Landau-Kleffner Syndrome (LKS), continuous spikes and waves during slow-wave sleep (CSWS), and atypical rolandic epilepsy (aRE) cases [13–15,29,38]. Monogenic mutations have been shown to cause epilepsy, especially when mutations occur in genes encoding ion channels [39].

We describe the functional effects of a *GRIN2A* missense mutation in the S2 region of the glutamate binding domain (LBD) of the GluN2A NMDA subunit found in three unrelated patients (Table 1). This amino acid change at residue 731 from aspartic acid to asparagine (D731N) results in the substitution of an uncharged amino acid for a charged amino acid. According to the crystal structure of the GluN2A [40,41], the residue Glu2A-D731 occupies a strategic position in close proximity to the glutamate molecule (Fig 1D). Functional analysis of GluN2A-D731N reveals a drastic decrease in glutamate potency (~3,000-fold). This likely reflects interactions (a water bridge) between the amino group of the agonist glutamate and the residue at position 731 [40,42]. The homologous positions of GluN2A-D731 were also reported to be the critical glycine binding positions in GluN1 [43]. In the GluN1 subunit, mutation of the homologous position GluN1-D732 to glutamate (D732E), asparagine (D732N), alanine (D732A), or glycine (D732G) decreased the potency of glycine by over 4,000-fold [43]. No response or very small responses to 10 mM glutamate and plus 10–30 mM glycine were observed in receptors harboring GluN2A-D731A, GluN2A-D731E, GluN2B-D732A, or GluN2B-D732E [42–44]. Mutation of Asp at position 731 may destabilize the glutamate binding pocket, resulting in a strong decrease of glutamate potency. Decreased potency for glutamate in the mutant receptor will reduce activation both at the synapse as well as for extrasynaptic NMDA receptors compared to the WT receptors. The decreased potency is also expected to accelerate the deactivation of the current responses following glutamate removal, and thus accelerate the EPSC time course [35, 45]. Interestingly, we also found that the mutant GluN2A-D731N also enhanced sensitivity to both zinc and protons, consistent with the strong coupling between downstream mechanisms that mediate proton and Zn^2+^ inhibition [46,47]. The enhanced sensitivity to negative allosteric modulators may reflect the enhanced sensitivity of channel opening in NMDARs harboring the D731N mutation to inhibition given the reduced open probability. However, further study is required to explain how the mutation in the agonist binding domain affects the zinc and proton inhibition. The increase in zinc and proton inhibition further diminishes activity of the mutant receptor through enhanced inhibition at physiological concentrations of these negative modulators. The combination of these two effects will strongly reduce NMDAR activation compared to WT GluN2A-containing NMDA receptors. Thus, our data suggest that the aspartic acid at position 731 residues is critical to NMDAR function.

Previously published reports suggest that epilepsy is associated with loss-of-function and gain-of-function GluN2A mutations [13,15,20–22,29,48], indicating that either enhanced or reduced NMDAR function could lead to epilepsy. In the brain, the neuronal network is constituted by excitatory neurons (as glutamatergic neurons) and inhibitory neurons (as GABAergic neurons). GluN2A/*GRIN2A* is expressed in both glutamatergic and GABAergic neurons of in the human fetal cerebral cortex [49] and in GABAergic interneurons in the prefrontal cortex [50]. Therefore, the alternation of functions and expression levels of GluN2A (caused by GluN2A mutations) in different neuron types may have different impacts on the balance of excitation and inhibition in brain circuits, as well as circuit development. This study indicates that loss-of-function GluN2A mutants may impair the inhibitory effect of GABAergic neurons and contribute to epilepsy.

The phenotype of all three unrelated patients was marked with developmental delay, particularly in language. All patients exhibited temporal lobe seizures. For at least one of the patients (in this study), EEG recordings confirmed seizure activity during sleep. Such encephalopathy with status epilepticus during sleep (ESES) has been shown to disrupt neural processes local to the site of activity [51]. Slow-wave activity during sleep is important in learning, with EEG activity correlated to similar areas in sleep and wakefulness [52]. All three probands experienced rolandic epilepsy from the temporal lobes, an area associated with language function. It is possible that the normal processes during sleep necessary for language development are perturbed by mutant NMDA receptor hypo-activity, disrupting normal circuitry. Further experimentation is required to validate this theory, but there is strong evidence for the importance in screening for NMDAR mutations in cases of idiopathic epilepsy. This study shows the functional effects of a *GRIN2A* mutation in a highly conserved portion of the ABD. Such work could ultimately lead to the identification of a new generation of drugs to mitigate or prevent the devastating effects of disorders such as LKS and other epilepsy-aphasia spectrum disorders.

Correlating phenotype to genotype is difficult because many genes may be involved, along with environmental factors, in expression of seizures. Gene sequencing technology may help to solve this problem, and has allowed us to identify multiple patients with an identical *GRIN2A* mutation. Functional analysis of *de novo* mutations verified through genomic sequencing in trios will aid in the understanding of certain types of epilepsy, which will ultimately lead to better treatment. Moreover, understanding the functional effect of mutations is clinically important, and may lead to better therapeutic solutions to their corresponding diseases.
